# High-resolution magic angle spinning NMR studies for metabolic characterization of *Arabidopsis thaliana* mutants with enhanced growth characteristics

**DOI:** 10.1371/journal.pone.0209695

**Published:** 2018-12-31

**Authors:** Dieuwertje Augustijn, Niels van Tol, Bert J. van der Zaal, Huub J. M. de Groot, A. Alia

**Affiliations:** 1 Leiden Institute of Chemistry, Leiden University, RA Leiden, The Netherlands; 2 Institute of Biology Leiden, Leiden University, BE, Leiden, The Netherlands; 3 Institute of Medical Physics and Biophysics, University of Leipzig, Leipzig, Germany; Ecole Normale Superieure, FRANCE

## Abstract

Developing smart crops which yield more biomass to meet the increasing demand for plant biomass has been an active area of research in last few decades. We investigated metabolic alterations in two *Arabidopsis thaliana* mutants with enhanced growth characteristics that were previously obtained from a collection of plant lines expressing artificial transcription factors. The metabolic profiles were obtained directly from intact *Arabidopsis* leaves using high-resolution magic angle spinning (HR-MAS) NMR. Multivariate analysis showed significant alteration of metabolite levels between the mutants and the wild-type Col-0. Interestingly, most of the metabolites that were reduced in the faster-growing mutants are generally involved in the defence against stress. These results suggest a growth-defence trade-off in the phenotypically engineered mutants. Our results further corroborate the idea that plant growth can be enhanced by suppressing defence pathways.

## Introduction

During the last few decades, the demand for agricultural products has increased dramatically [1–3]. In order to meet the actual food demand in 2050, a 70% increase of the food production has to be realized in the coming three decades [4,5]. A possible way to meet this demand is to develop smart crops, varieties which can give more yield with fewer inputs [5,6]. This would also reduce the need for chemicals such as pesticides and fungicides.

New crop varieties with improved agronomic traits can be developed by traditional breeding methods [7], recently aided by the use of new genome-editing technologies such as provided by site-specific nucleases as CRISPR/CAS [8,9]. Recently, we have explored genome interrogation using zinc finger artificial transcription factors (ZF-ATFs) as a novel technique to drastically modify genome-wide transcription patterns and to generate novel phenotypes of interest in the model plant species *Arabidopsis thaliana* [9–12]. In these studies, arrays of three zinc fingers (3F) were fused to the transcriptional activation domain of the VP16 protein of the herpes simplex virus [10]. Any 3F motif can recognize 9 base pairs of DNA, corresponding to approximately 1000 recognition sites in the nuclear *Arabidopsis* genome. Expression of a single 3F-VP16 fusion under control of the meristematic *RPS5A* promoter can thus lead to transcriptional activation of a large number of genomic loci, and consequently to drastic metabolic and phenotypic changes [9,11]. Previously, we have screened a population of transgenic Arabidopsis plants harbouring 3F-VP16 encoding gene constructs using enhanced rosette surface area (RSA) as a selection criterion for enhanced overall biomass accumulation [9,12]. From this phenotypic screen, we isolated two novel mutants designated VP16-02-003 and VP16-05-014 with respectively a 55% and 33% significantly larger RSA compared to the wild-type Col-0, each expressing a specific 3F-VP16 fusion protein. The growth differences did not relate to a differential development in the mutants as compared to the wild-type Col-0 [12]. In that previous study, a transcriptomics analysis was also performed to investigate the changes in the gene expression patterns [12]. Interestingly, we observed an overlap in the transcriptional changes in that correlated with the increase in RSA. Most notably, shared downregulated genes were found to be involved in several defence processes, including response to stress (GO:0006950), response to external stimulus (GO:0009605), response to wounding (GO:0009611), response to endogenous stimulus (GO:0009719), response to jasmonic acid (GO:0009753), response to stimulus (GO:0050896), defence response by cell wall thickening (GO:0052482) and defence response by callose deposition in cell wall (GO:0052544) (S1 Table) [12].

For a comprehensive understanding of newly developed plant genotypes, a systems biology approach is indispensable. Using this approach, a plant is seen as a system of interacting units that can be analysed as a whole rather than focusing on individual changes [6,13]. One of the system biology approaches is the metabolomics approach, which aims to determine small molecules that are involved in various physiological functions, such as growth, productivity and defence [14]. Directly examining the metabolic profiles of intact *Arabidopsis* leaves without any extraction is important to understand the functional framework of metabolism in the leaves. Recently, we have established high-resolution magic angle spinning nuclear magnetic resonance (HR-MAS NMR) to obtain the metabolic profile directly from intact wild-type *Arabidopsis* leaves [15].

In this study, we applied one- and two-dimensional HR-MAS NMR to obtain the metabolic profile directly from the intact leaves of wild-type Columbia (Col-0) *Arabidopsis* plants, and of the VP16-02-003 and VP16-05-014 mutants with enhanced growth characteristics and putatively higher sensitivity to biotic stress based on transcriptomics data. Through metabolic profiling in the native state in combination with multivariate analysis, we here provide novel insights into the biochemical pathways correlated to the enhanced rosette surface area phenotype for both mutants.

## Materials & methods

### Plant materials

*Arabidopsis thaliana* plants were grown in soil and cultivated in a growth chamber maintained at 293 K, 70% relative humidity and at a 12 h light (200 μmol m^-2^ s^-1^ photosynthetically active radiation) and 12 h dark regime [15]. Experiments were performed using the *Arabidopsis thaliana* accession Columbia-0 (Col-0) as wild-type. The VP16-02-003 and the VP16-05-014 mutant (both T3 generation) with an increased rosette surface area were obtained by phenotypic screening of a population of transgenic Arabidopsis plants harbouring 3F-VP16 encoding T-DNA constructs, as described previously [12]. The larger rosette surface area of both mutants are confirmed using ImageJ [12].

### Quantification of free amino acids, soluble sugars, proteins and starch

The soluble sugar content was determined using the phenol-sulphuric acid method at a wavelength of 490 nm [16]. Glucose concentrations ranging from 0 to 250 μg/mL were used to obtain a standard curve. The free amino acids content was determined using the ninhydrin method as described previously [17]. Proteins were extracted from the leaves as described before [18]. Protein content was quantified by a Bradford assay [19]. The starch content of the leaves was measured by determining the glucose released with α-amylase and amyloglucosidase as described by Smith and Zeeman [20].

### Statistical analysis

The data for each independent experiment were subjected to the Student’s t-test. The OriginPro 2016 software (Northampton, USA) was used to determine the differences between Col-0 and the VP16-02-003 and VP16-05-014 mutants. Values are presented as means ± standard error (SEM) and statistical significance was determined at p < 0.05.

### HR-MAS NMR-based metabolic profiling

The leaves were harvested from the plants at 28 days after germination (dpg, growth stage 3.70–3.90), frozen immediately in liquid nitrogen and stored at -80°C until use. A single rosette leaf (0.0684 ± 0.0087 mg, 8 different plants for every genotype) was inserted into a 4 mm zirconium oxide (ZrO_2_) rotor. 10 μl of deuterated phosphate buffer (100 mM, pH 6) containing 0.1% (w/v) 3-trimetylsilyl-2,2,3,3-tetradeuteropropionic acid (TSP) was added as a lock solvent and NMR reference. ^1^H High-Resolution Magic Angle Spinning (HR-MAS) NMR experiments were performed with a Bruker DMX 400 MHz spectrometer operating at a resonance frequency of 399.427 MHz. The instrument is equipped with a 4 mm HR-MAS dual inverse ^1^H/^13^C probe with a magic angle gradient. Data were collected with a spinning frequency of 4 kHz at a temperature of 277 K.

The one-dimensional ^1^H HR-MAS NMR spectra were recorded using a rotor synchronized Carr-Purcell-Meiboom-Gill (CPMG) pulse sequence with water suppression [21]. Each one-dimensional spectrum was acquired applying 256 transients, a spectral width of 8000 Hz, a data size of 16 K points, an acquisition time of 2 seconds and a relaxation delay of 2 seconds. The free induction decays (FIDs) were exponentially weighted with a line broadening of 1 Hz. Spectra were phased manually and automatically baseline corrected using TOPSPIN 2.1 (Bruker Analytische Messtechnik, Germany). A gradient-enhanced two-dimensional ^1^H-^1^H-COSY sequence was applied in order to confirm signal assignments as described before [14].

### Multivariate analysis

A bucket table was generated from the one-dimensional spectra using AMIX software (version 3.8.7, BrukerBioSpin). The region between 4.20–6.00 ppm was excluded from the analysis to remove the large water signal. The one-dimensional CPMG spectra were normalized to the total intensity and binned into buckets of 0.04 ppm. The data was mean centred and the Pareto scaling method was used [22]. Unsupervised Principal Component Analysis (PCA) and supervised Orthogonal Projections to Latent Structures Discriminant Analysis (OPLS-DA) were performed on the bucket table using the SIMCA software package version 14.0 (Umetrics, Umeå, Sweden). The quality of these models was evaluated by the R^2^X and R^2^Y, the goodness-of-fit parameters, and Q^2^, a measure of the quality of the model based on cross-validation [23,24]. One sample from the VP16-05-014 dataset was removed as it was a significant outlier defined as an observation located outside the 95% confidence region of the Hotelling’s T2 ellipse in the PCA scatter plot (see Supplementary S1 Fig) [25]. Further analysis was performed without this outlier. OPLS-DA was used to determine the buckets which are different between the mutants and the wild-type Col-0. In addition, two OPLS-DA models were constructed for each mutant; Col-0 vs VP16-02-003 and Col-0 vs VP16-05-014 (S2 Fig). The shared and unique structures between these two OPLS-DA models were investigated using a SUS (shared and unique structures) plot [26].

### Biomarker identification

The metabolites corresponding to the peaks of interest in the determined buckets were identified by the Biological Magnetic Resonance Data Bank (BMRB) (www.bmrb.wisc.edu/metabolomics), Platform for RIKEN Metabolomics [27,28] and Chenomx NMR Suite 8.2 (Chenomx Inc., Edmonton, Alberta, Canada).

### Quantification of metabolites

Chenomx NMR Suite 8.2 (Chenomx Inc., Edmonton, Alberta, Canada) was used for quantitative NMR data analysis. The concentrations of the various metabolites in the spectra of the leaves from wild-type and mutant *Arabidopsis* were determined by the known concentration of the reference peak of TSP. All Student’s t-test analyses of the NMR quantification results were performed with OriginPro 2016 (Northampton, USA), The t-test is used after multivariate analysis (where correlation between metabolites was already taken into account).

## Results and discussion

**Fig 1:**
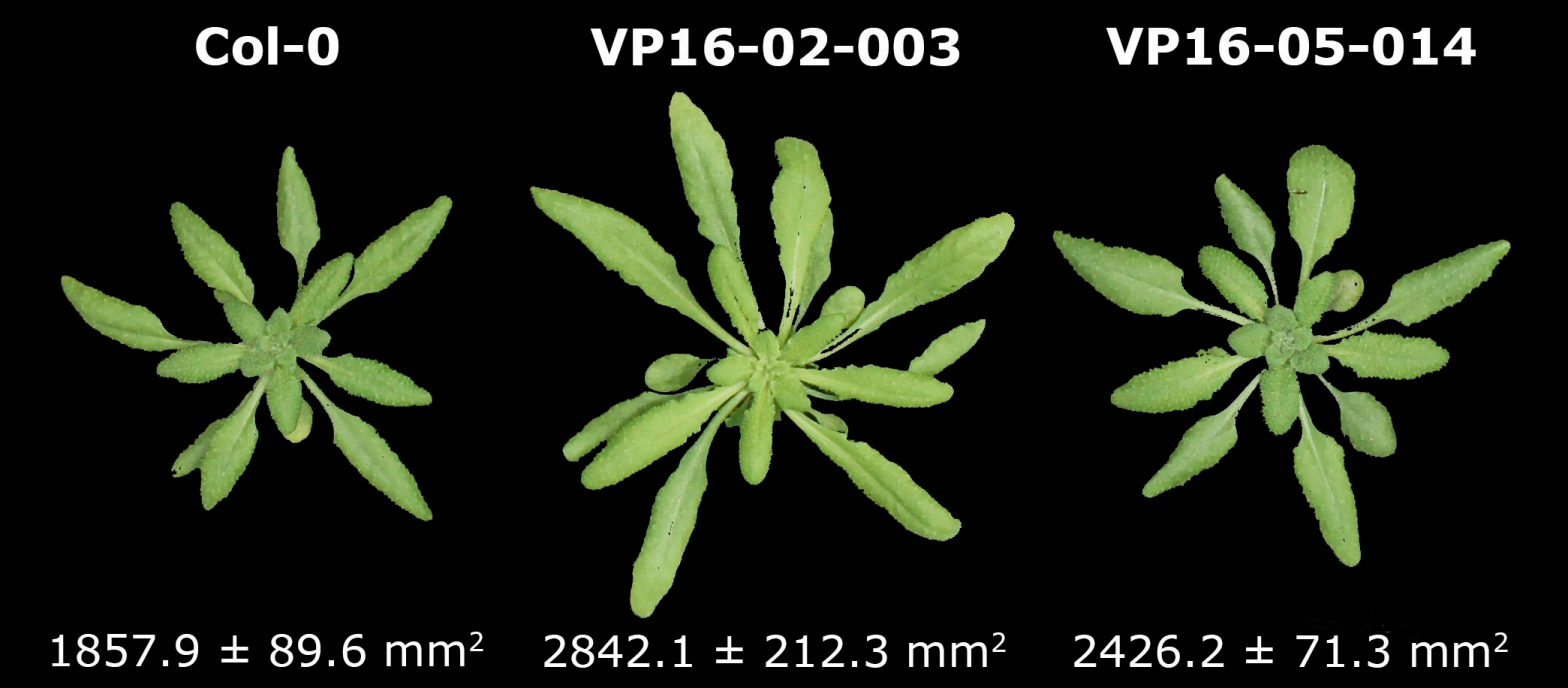
Representative overview of the rosette phenotypes of Arabidopsis Col-0, VP16-02-003 and VP16-05-014 at 28 days post germination. Data of average rosette surface area is shown below each images as mean ± SEM (n = 18).

The larger rosette surface area phenotype of the VP16-02-003 and VP16-05-014 mutant was confirmed by determining the rosette surface area (RSA) at 28 days post germination (Fig 1). The VP16-02-003 and the VP16-05-014 mutant have respectively 53% and 31% larger RSA in comparison to Col-0. This is in line with our earlier publication on these *Arabidopsis* mutants [12].

### Levels of free amino acids, soluble sugars, proteins and starch

Prior to metabolic profiling, the concentration of free amino acids, proteins, soluble sugars and starch was determined for extracts of leaves from the VP16-02-003 and VP16-05-014 mutant and Col-0 to get a broader overview of amino acids, sugars, protein and starch. Interestingly, an overall decline in free amino acids, soluble sugars, proteins, as well as starch content, was observed for the VP16-02-003 and VP16-05-014 mutants of *Arabidopsis* as compared to Col-0 (Table 1).

**Table 1:** Total free amino acids, protein, soluble sugar and starch content in mg/g fresh weight for leaves of *Arabidopsis thaliana* Col-0, VP16-02-003 and VP16-05-014.

Content (mg/g FW)	Col-0	VP16-02-003	VP16-05-014
Free amino acids	1.48 ± 0.05	1.17 ± 0.05 *	1.04 ± 0.01 *
Protein	1.57 ± 0.06	1.34 ± 0.08 *	0.98 ± 0.01 *
Soluble sugar	0.18 ± 0.01	0.11 ± 0.01 *	0.13 ± 0.01 *
Starch	0.57 ± 0.02	0.23 ± 0.01 *	0.31 ± 0.02 *

Data is expressed as mean ± SEM (n = 5).

* p < 0.05 compared with Col-0

Sulpice *et al*. observed a negative correlation between biomass and the levels of starch, total protein and total free amino acids in *Arabidopsis* [29]. The level of soluble sugars such as sucrose has also found to be negatively correlated with biomass [29]. Sugars, such as glucose and sucrose are important products of photosynthesis and play an essential role in controlling plant growth, development and defence [14]. The decline in the level of soluble sugars accompanied by an increase in overall biomass and no significant difference in development of the two mutants indicates that sugar resources are likely diverted toward growth and storage products in these mutants, rather than defence-related processes.

### Metabolic profiling of the VP16-02-003 and VP16-05-014 mutant

**Fig 2:**
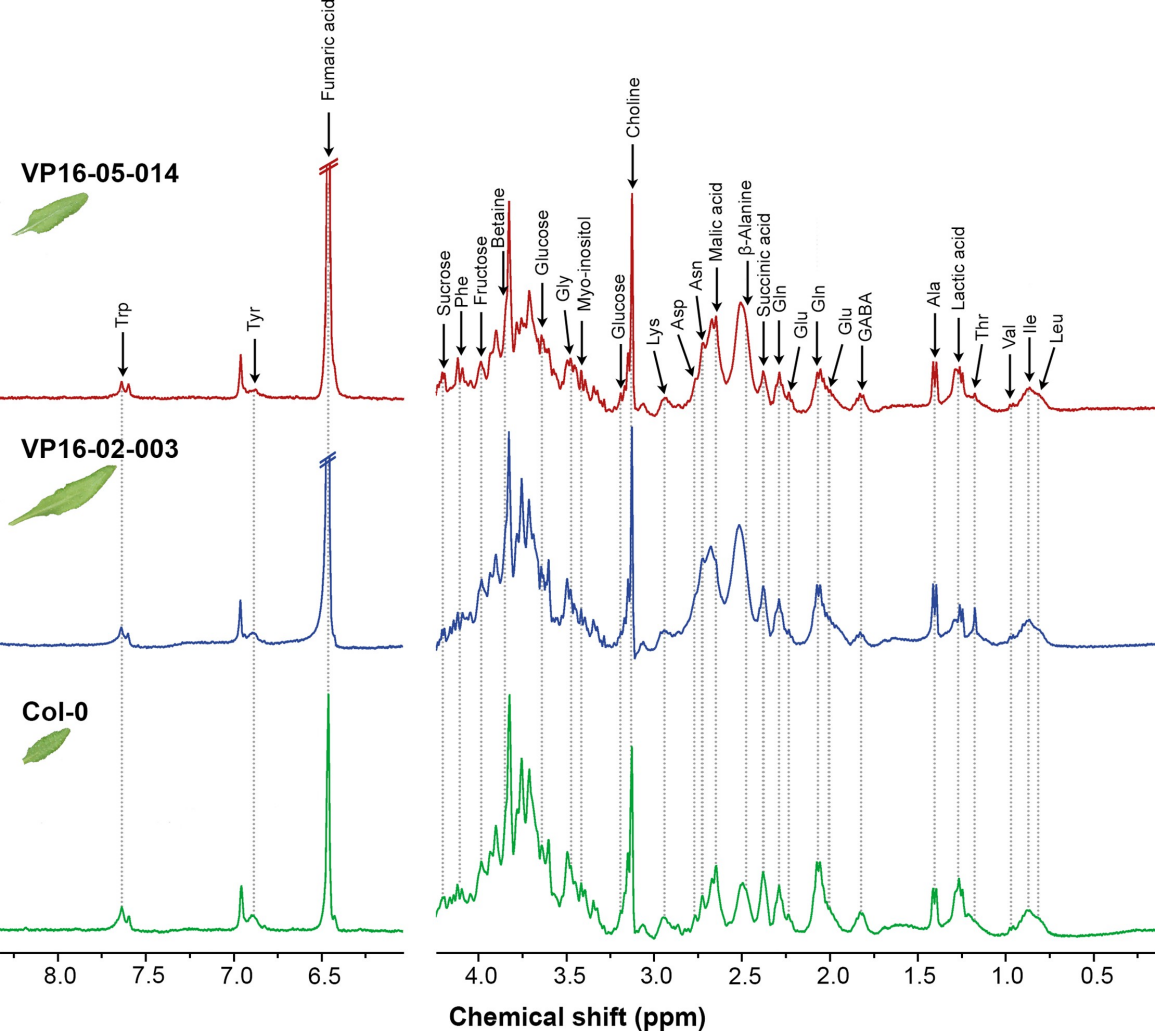
Representative one-dimensional ^1^H HR-MAS NMR spectra collected from intact leaves of *Arabidopsis thaliana* Col-0 (bottom panels), VP16-02-003 (middle panels) and VP16-05-014 (top panels) grown in a 12h-light/12h-dark regime. The intact leaves were harvested 28 dpg at t = 6 hours (6 hours after the beginning of the light period) for the measurement. The signals from the assigned metabolites have been shown in the spectra (see S2 Table for assignment).

In order to identify metabolites and biochemical pathways responsible for the increased rosette surface area shared phenotype of the VP16-02-003 and VP16-05-014 mutant, their metabolic profiles have been analysed for intact leaves using HR-MAS NMR. In Fig 2, representative one-dimensional ^1^H NMR spectra of Col-0, VP16-02-003 and VP16-05-014 are shown. Two-dimensional homonuclear correlation spectroscopy (^1^H-^1^H COSY) enabled confirmation of metabolites by their spin systems. In addition, the 2D ^1^H-^1^H COSY data can be used to validate changes observed in the 1D spectral envelope for the mutants as compared to Col-0. The signals of various metabolites were assigned with the help of literature data from the Biological Magnetic Resonance Data Bank (BMRB) [30,31]. S2 Table shows a list of identified metabolites.

### Identification of the increased rosette surface area shared phenotype

**Fig 3:**
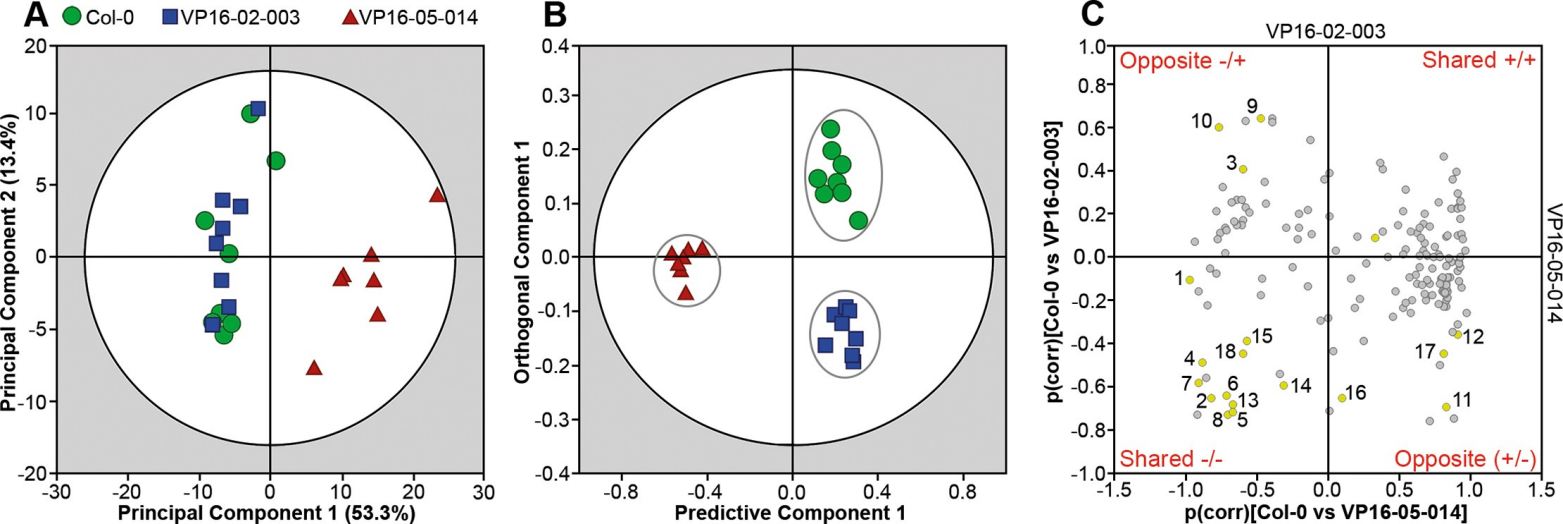
Multivariate analysis of ^1^H HR-MAS NMR metabolic data collected from *Arabidopsis thaliana* Col-0 (●), VP16-02-003 (■) and VP16-05-014 (▲). (A) PCA score plot with R^2^X = 0.761 and Q^2^ = 0.611. The dark circle shows the 95% confidence interval using Hotelling T^2^ statistics. (B) OPLS-DA score plot with R^2^X = 0.849, R^2^Y = 0.942, Q^2^ = 0.603, which indicates separation between the Col-0, VP16-02-003 and VP16-05-014 *Arabidopsis* plants based on their metabolic profile. The dark circle represents the Hotelling T^2^ interval with 95% confidence. (C) SUS plot represents biomarkers responsible for the separation in the score plot. 1. Fumaric acid; 2. Malic acid; 3. Lactic acid; 4. Fructose; 5. Glucose; 6. Myo-inositol; 7. Choline; 8. Betaine; 9. L-alanine; 10. β-alanine; 11. L-asparagine; 12. L-aspartic acid; 13. L-glutamic acid; 14. L-glutamine; 15. L-glycine; 16. L-lysine; 17. L-phenylalanine; 18. L-tyrosine.

To probe if *Arabidopsis thaliana* Col-0, VP16-02-003 and VP16-05-014 can be discriminated from each other based on their metabolic profiles, multivariate analysis was applied to the HR-MAS spectra from both mutants and Col-0. Unsupervised PCA was performed which explained 76.1% of the variation by a three-component model (see Fig 3A), which shows a clear group separation of VP16-05-014 from the Col-0 and VP16-02-003. In contrast, there was no clear group separation between Col-0 and VP16-02-003 in the PCA score plot. Supervised OPLS-DA was applied to further understand the separation between the wild-type Col-0, VP16-02-003 and VP16-05-014 and to identify crucial candidates biomarkers involved in the increased rosette surface area phenotype. Fig 3B shows the score plot of the OPLS-DA. The R^2^X, R^2^Y and Q^2^ were 0.849, 0.942 and 0.603, respectively. The OPLS-DA model was found to be of good quality and has an accurate prediction. The score plot also shows that the biological variation for the VP16-02-003 or VP16-05-014 is less than for the wild-type *Arabidopsis* Col-0.

A Shared and Unique Structures (SUS) plot can be a powerful method to identify potential biomarkers for the enhanced growth characteristics for both mutants [26]. To obtain a SUS plot, two separate OPLS-DA models (Col-0 versus VP16-02-003 and Col-0 versus VP16-05-014) were generated from the metabolic profiles (S2 Fig). The correlation coefficients of the predictive component p(corr) for both models are plotted against each other in Fig 3C. Concentrations of metabolites plotted in the upper right corner of the SUS-plot increased and those plotted in the lower left corner decreased in both mutants as compared to Col-0. The upper left corner and lower right corner of the SUS-plot contain metabolites with anticorrelated concentrations in the two mutants in comparison to Col-0. Eighteen biomarkers were determined from the SUS plot which show variation between Col-0, VP16-02-003 and VP16-05-014 mutants, including organic acids (fumaric acid, malic acid, lactic acid), sugars (fructose, glucose), a sugar alcohol (myo-inositol), precursor of cell wall components (choline), an organic osmolyte (betaine) and free amino acids (L-alanine, β-alanine, L-asparagine, L-aspartic acid, L-glutamic acid, L-glutamine, L-glycine, L-lysine, L-phenylalanine and L-tyrosine). Most of the identified biomarkers are primary metabolites directly involved in essential processes as growth, development and defence [32,33]. This provides evidence that the larger rosette surface area phenotype of both mutants involves re-allocation of primary resources.

### Metabolic evidence for altered growth-defence trade-off in the VP16-02-003 and VP16-05-014 mutants

**Fig 4:**
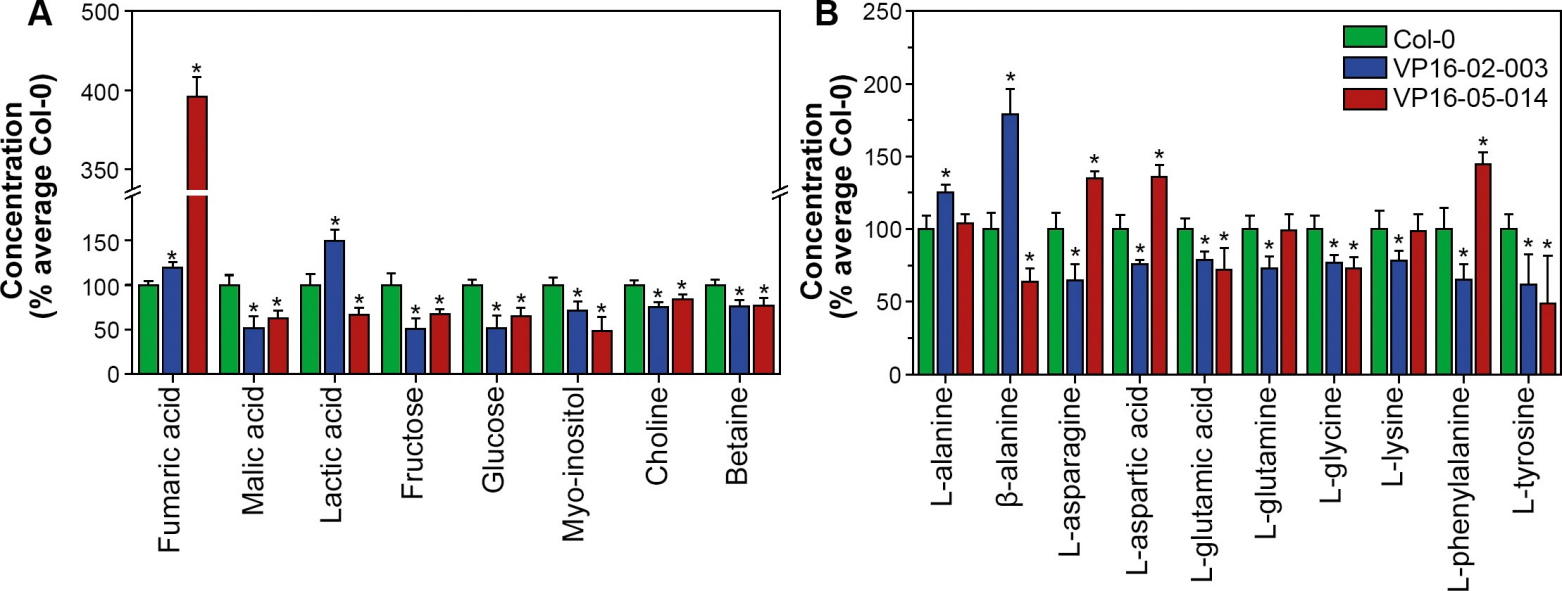
Metabolic alterations in the leaf of *Arabidopsis thaliana* VP16-02-003 and VP16-05-014 plants as compared to Col-0 plants grown in a 12h-light/12h-dark regime by ^1^H HR-MAS NMR. (A) Relative levels of organic acids, sugars, sugar alcohol, precursor of cell wall components and organic osmolyte in leaves of VP16-02-003 and VP16-05-014 plants in comparison to the level in Col-0. (B) Relative levels of free amino acids in leaves of VP16-02-003 and VP16-05-014 plants in comparison to Col-0. Concentrations are represented by the mean ± SEM averaged over n = 8 samples. The asterisks (*) indicate significant differences between concentrations for Col-0 and mutants, calculated with Student’s t-test (p < 0.05).

The quantitative analysis of the metabolites that show significant variation between Col-0, VP16-02-003 and VP16-05-014 plants is shown in Fig 4.

Organic acids, like fumaric acid and malic acid, play an important role in the major carbon metabolism involving glycolysis, the tricarboxylic acid (TCA) cycle, and the photorespiration cycle [34,35]. The fumaric acid level was significantly elevated by 19.5% in the VP16-02-003 mutant and by 295.8% in the VP16-05-014 mutant (Fig 4A). The primary metabolite fumaric acid participates in multiple pathways in plant metabolism and is considered to be one of the major forms of fixed carbon in some C3 plants, including *Arabidopsis* [15,36]. In particular, fumaric acid can accumulate to levels of several milligrams per gram fresh weight in *Arabidopsis* leaves, often exceeding concentrations of starch and soluble sugars [36].

In contrast to fumaric acid, the malic acid level is reduced for both mutants (Fig 4A). Malic acid is involved in various physiological functions in the plant cells, such as supplying NADH for nitrate reduction and carbon skeletons and delivering NADPH for fatty acid biosynthesis [37]. The observed reduced malic acid level for both mutants can be a consequence of its enhanced utilization in downstream pathways involved in the growth promoting phenotype [38]. The role of lactic acid in the leaves of *Arabidopsis thaliana* is not very clear. It has been reported to play a role in plant defence against pathogens [38]. Also, a growth promoting effect of lactic acid has been reported earlier [39,40]. In our study, no common trend was observed in the levels of lactic acid in two mutants. The lactic acid concentration was elevated by 49.1% in the VP16-02-003 mutant (p < 0.05), and reduced by 33.2% in the VP16-05-014 mutant (p < 0.05) (Fig 4A). Thus no growth promoting effect of lactic acid can be generalized from our study.

Primary sugars in plants such as glucose, fructose, and sucrose, are produced during photosynthesis, provide the primary energy supply and serve as storage metabolites in plants. These sugars have a regulatory role in photosynthesis, growth and development and as a signalling molecule to modulate gene expression [41]. The levels of fructose and glucose were in both the VP16-02-003 and the VP16-05-014 mutants significantly decreased in comparison to Col-0 (Fig 4A). Decrease in glucose level is also in line with downregulation of glucan 1,3-beta-glucosidase (AT1G64760) seen in transcriptome data of these two mutant (S1 Table) [12]. Glucan endo-1,3-beta-glucosidase is involved in carbohydrate metabolism and has been shown to be linked with plant defence to fungus and nematodes [42]. Although the stress response is a very dynamic process and differs for every stress type, soluble sugar concentrations are altered during defence and strongly decrease in response to different forms of abiotic stress, as energy is needed to operate defence mechanisms. In general low sugar concentrations lead to an impaired abiotic and biotic stress response [41,43].

Myo-inositol is a signalling metabolite in *Arabidopsis thaliana* [44]. It is involved in stress response, regulation of cell death and cell wall biosynthesis [45,46]. The concentration of myo-inositol was reduced by 28.3% (p < 0.05) in the VP16-02-003 mutant and by 51.7% in the VP16-05-014 mutant (p < 0.05) (Fig 4A). A reduced pool of myo-inositol in the VP16-02-003 and VP16-05-014 mutant may reflect an impaired defence pathway regulation in these mutants. For instance, in a previous study, the reduced level of myo-inositol was observed in the *mips1* mutant, a mutant which shows increased sensitivity to reactive oxygen species stress [45].

Choline is an important precursor for membrane phospholipids in plants. Choline can be oxidized in a 2-step reaction via betaine aldehyde to betaine (glycine betaine, *N*,*N*,*N-*trimethylglycine). Accumulation of betaine in *Arabidopsis thaliana* leads to a higher tolerance for abiotic stress [47,48]. The choline and betaine levels in the VP16-02-003 and the VP16-05-014 mutant were both significantly reduced in comparison to the wild-type Col-0 (Fig 4A). This is in line with less available resources for stress resistance and defence and low concentrations of the soluble primary carriers malic acid, fructose and glucose, as well as more storage in the form of fumaric acid.

Amino acids are essential precursors for a wide range of cellular components like proteins, nucleotides, chlorophylls and nitrogen-containing compounds [49,50]. Fig 4B shows the concentrations of free amino acids in the VP16-02-003 and the VP16-05-014 mutant in comparison to *Arabidopsis* Col-0. In both VP16-02-003 and VP16-05-014 mutants, levels of L-glutamic acid, L-glycine and L-tyrosine were decreased relative to Col-0. This decline is consistent with overall decrease in total free amino acids shown in Table 1. The pattern of decline in these amino acids may be associated with decrease in the level of sugars. From earlier studies, it is known that a decreased level of sugar leads to the inhibition of amino acid biosynthesis [49]. Since, amino acid metabolism plays a regulatory role in the response to stress [50][51], a decline in the levels of these free amino acids for both mutants may reflects their reduced investments to defence responses against stress. A decline in glycine in both mutants may be linked with downregulation of Glycine-rich RNA binding proteins observed in transcriptome data of these mutants. Glycine-rich proteins are known to be involved in plant stress responses [52]. Downregulation of Glycine-rich RNA binding proteins together with low glycine levels thus signify low defence response of VP16-02-003 and VP16-05-014 mutant. The levels of other free amino acids such as L-asparagine, L-aspartic acid, L-glutamine, L-lysine and L-phenylalanine were also lower in VP16-02-003 mutant in contrast to Col-0. However, their levels either did not change or increased in VP16-02-003 mutant with respect to Col-0. The reason for the differences in some of the amino acid pattern in two mutants is presently not understood. Interestingly the levels of L-alanine and β-alanine were significantly higher in the VP16-02-003 mutant in comparison to the wild-type Col-0. An increase in alanine under sulphur limitation has been reported earlier [53]. Transcriptome analysis of this mutant has revealed downregulation of sulphate metabolism genes [12]. High levels of alanine in the VP16-02-003 mutant may thus be connected to reduced sulphur fixation.

**Fig 5:**
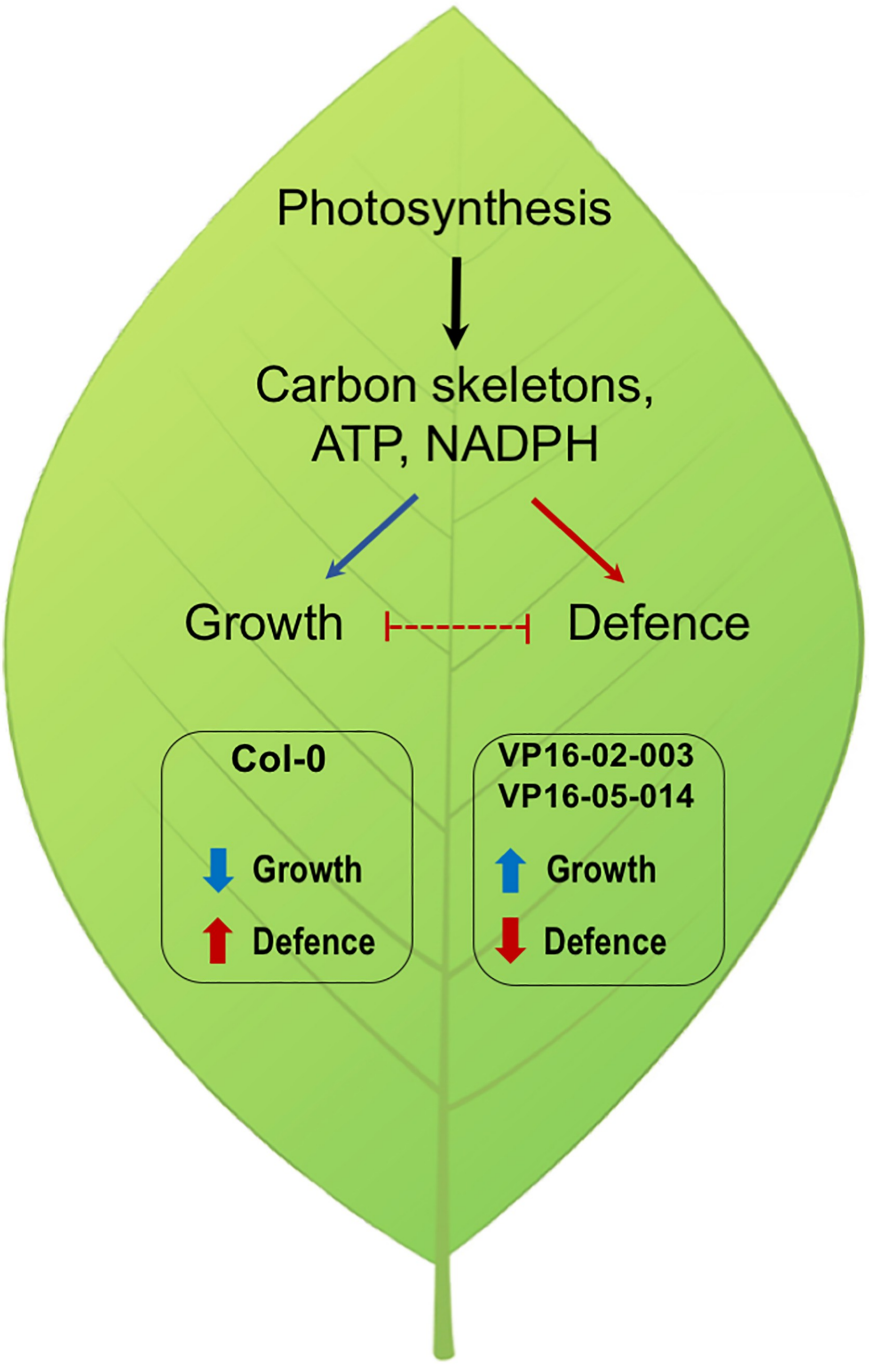
Proposed model for the different growth-defence trade-off in the VP16-02-003 and VP16-05-014 mutant with a larger rosette surface area shared phenotype in comparison to wild-type *Arabidopsis thaliana* Col-0.

The changes of metabolic profiles for the VP16-02-003 and the VP16-05-014 mutant that share a larger rosette surface area phenotype are mostly associated with the response to stress. These results are in line with our earlier transcriptome analysis, which shows that many genes that are downregulated in both mutants are involved in defence processes [12]. Recently, Fusari et al. [54] have investigated the genetic architecture of central metabolism by mapping metabolite quantitative trait loci (QTL). The results of genome-wide associated mapping clearly revealed a well-defined trade-off between growth and defence in *Arabidopsis* which involve a fine-tuning of central metabolism. Thus, adaptation of the physiological function in *Arabidopsis thaliana* requires balancing of primary metabolites [55]. The trade-off between plant growth and defence implies that both are negatively correlated [55–57]. Hence the growth will improve when the defence system is less active in plants. Our results show that the VP16-02-003 and the VP16-05-014 mutants have a reduced defence response against stress (Fig 5), which shifts the balance toward enhanced growth which involves changes in metabolism. Some of the recent leaf and apical damage studies, show mixed results on whether a trade-off exist between growth, tolerance and defence [57–61]. Thus, future studies on damaged and undamaged states of VP16-02-003 and VP16-05-014 mutants will be important to dissect out the mechanistic insight into growth-defence conflict.

The fundamental understanding about linking the gene regulatory network to the physiological response obtained in this work paves the way for further investigations. In particular, it is known that physiological functions in *Arabidopsis* are regulated by the circadian cycle [62,63] and analysis of rhythmic patterns of the biomarkers in the VP16-02-003 and the VP16-05-014 mutants may help to resolve the underlying mechanisms involved in growth-defence trade-off.

## Conclusion

In this study, the metabolic profiles of the *Arabidopsis thaliana* mutant lines VP16-02-003 and VP16-05-014 with a larger rosette surface were further phenotyped and also investigated with the HR-MAS NMR-based metabolomics approach. The results provide converging evidence that the alteration of the metabolic profile of both mutants is due to lower defence responses against stress. Growth-defence trade-offs will thus have to be acknowledged for when trying to generate crops with improved growth characteristics.

## Supporting information

S1 Fig Principal component analysis (PCA) score plots derived from one-dimensional ^1^H HR-MAS NMR spectra of *Arabidopsis thaliana* Col-0 (●), VP16-02-003 (■) and VP16-05-014 (▲) with the outlier for VP16-05-014. A three components model with R^2^X = 0.756, Q^2^ = 0.585. The dark ellipse represents the Hotelling T^2^ interval with 95% confidence.
(TIF)

S2 Fig **Score plot of the orthogonal partial least square-discriminant (OPLS-DA) model derived from ^1^H HR-MAS spectra of *Arabidopsis thaliana* Col-0 and VP16-02-003 (A) and Col-0 and VP16-05-014 (B).** For model A: R2X = 0.865, R2Y = 0.999, Q2 = 0.568. For model B: R2X = 0.666, R2Y = 0.975, Q2 = 0.933. The dark ellipse shows the 95% confidence interval using Hotelling T2 statistics.
(TIF)

S1 Table Downregulated genes in the VP16-02-003 and VP16-05-014 mutant and the related GO terms referred to stress or defence. (DOCX)

S2 Table Measured metabolites in leaves of Col-0, VP16-02-003 and VP16-05-014 plants and their chemical shift assignment in 1H HR-MAS NMR spectrum. (DOCX)
