# Supplementary figures and images for: High-resolution magic angle spinning NMR studies for metabolic characterization of Arabidopsis thaliana mutants with enhanced growth characteristics
Source: PLoS One. 2018 Dec 31;13(12):e0209695. doi: 10.1371/journal.pone.0209695 (PMC6312362; doi:10.1371/journal.pone.0209695)

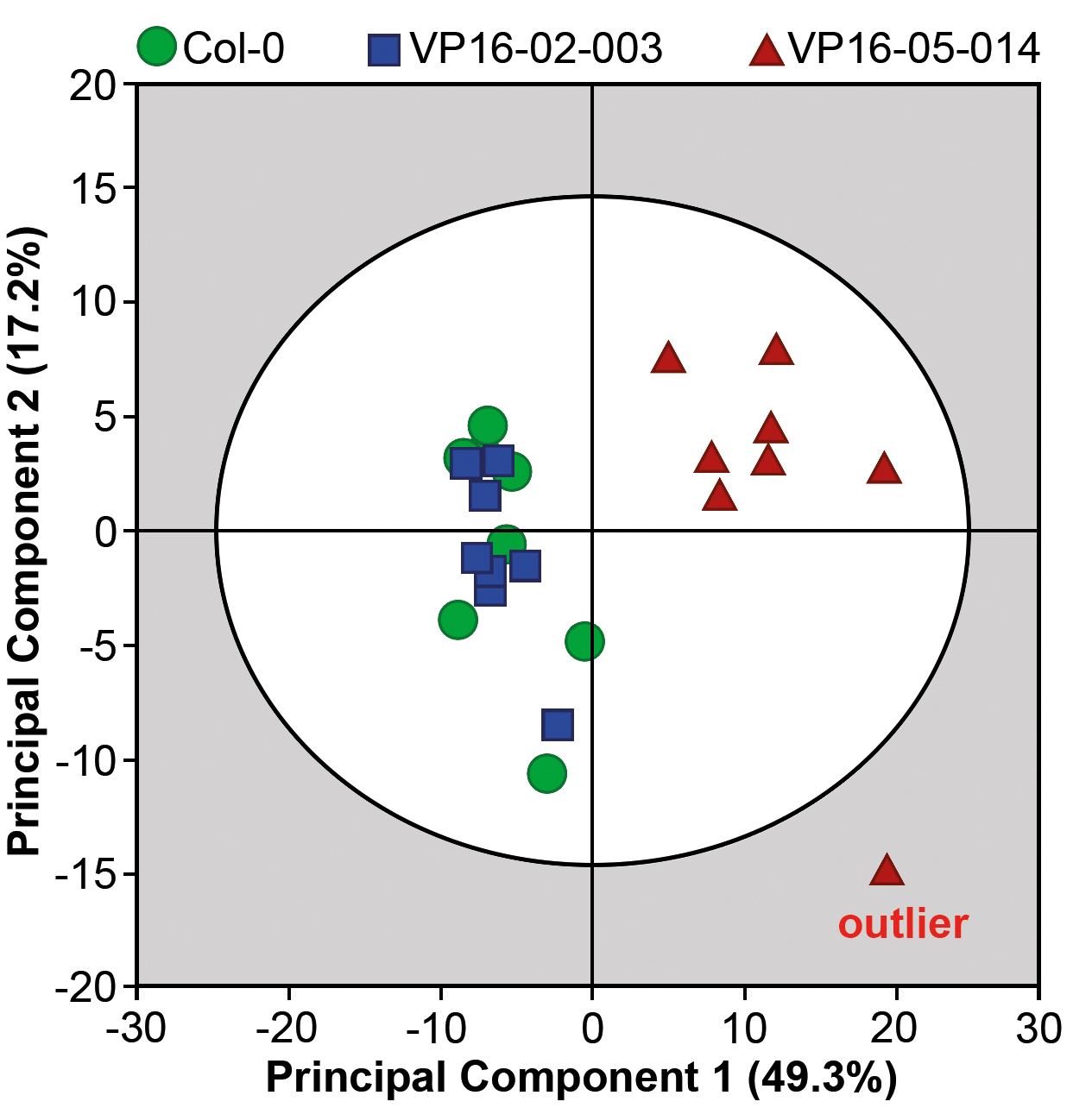

Supplement: S1 Fig — A three components model with R2X = 0.756, Q2 = 0.585. The dark ellipse represents the Hotelling T2 interval with 95% confidence. (TIF) [file pone.0209695.s001.tif]

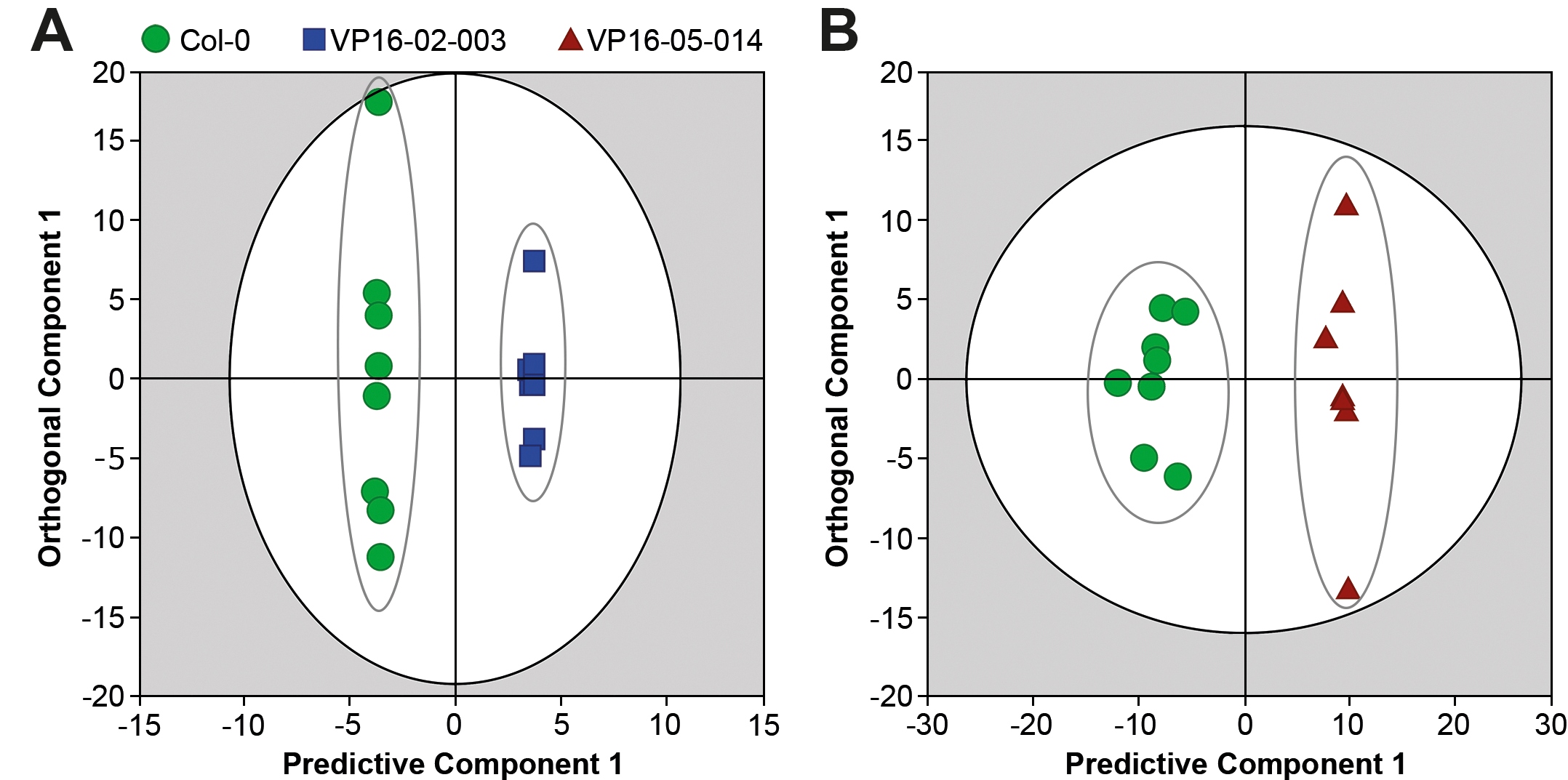

Supplement: S2 Fig — Score plot of the orthogonal partial least square-discriminant (OPLS-DA) model derived from 1H HR-MAS spectra of Arabidopsis thaliana Col-0 and VP16-02-003 (A) and Col-0 and VP16-05-014 (B). For model A: R2X = 0.865, R2Y = 0.999, Q2 = 0.568. For model B: R2X = 0.666, R2Y = 0.975, Q2 = 0.933. The dark ellipse shows the 95% confidence interval using Hotelling T2 statistics. (TIF) [file pone.0209695.s002.tif]
