# Supplementary material for: High-resolution magic angle spinning NMR studies for metabolic characterization of Arabidopsis thaliana mutants with enhanced growth characteristics
Source: PLoS One. 2018 Dec 31;13(12):e0209695. doi: 10.1371/journal.pone.0209695 (PMC6312362; doi:10.1371/journal.pone.0209695)
Supplement: S1 Table — (DOCX) [file pone.0209695.s003.docx]

**S1 Table: Downregulated genes in the VP16-02-003 and VP16-05-014 mutant and the related GO terms referred to stress or defence**

| **Gene** | **Description** | **GO terms** |
| --- | --- | --- |
| AT1G11670 | Protein DETOXIFICATION 36 (DTX36) | Response to stimulus (GO:0050896) |
| AT1G13930 | At1g13930/F16A14.27 | Response to stress (GO:0006950)  Response to stimulus (GO:0050896) |
| AT1G18710 | MYB transcription factor (AtMYB47) | Response to stress (GO:0006950)  Response to endogenous stimulus (GO:0009719)  Response to jasmonic acid (GO:0009753)  Response to stimulus (GO:0050896) |
| AT1G20030 | Pathogenesis-related thaumatin superfamily protein | Response to stimulus (GO:0050896) |
| AT1G20510 | 4-coumarate--CoA ligase-like 5 (4CLL5) | Response to stress (GO:0006950)  Response to external stimulus (GO:0009605)  Response to wounding (GO:0009611)  Response to stimulus (GO:0050896) |
| AT1G22770 | Protein GIGANTEA (GI) | Response to stress (GO:0006950)  Response to stimulus (GO:0050896) |
| AT1G24100 | Glycosyltransferase (UTG74B1) | Response to stress (GO:0006950)  Response to stimulus (GO:0050896) |
| AT1G32640 | ZBF1 | Response to stress (GO:0006950)  Response to external stimulus (GO:0009605)  Response to wounding (GO:0009611)  Response to endogenous stimulus (GO:0009719)  Response to jasmonic acid (GO:0009753)  Response to stimulus (GO:0050896) |
| AT1G61890 | Protein DETOXIFICATION 37 (DTX37) | Response to stimulus (GO:0050896) |
| AT1G70700 | TIFY domain/Divergent CCT motif family protein (JAZ9) | Response to endogenous stimulus (GO:0009719)  Response to jasmonic acid (GO:0009753)  Response to stimulus (GO:0050896) |
| AT1G72520 | Lipoxygenase 4, chloroplastic (LOX4) | Response to stress (GO:0006950)  Response to external stimulus (GO:0009605)  Response to wounding (GO:0009611)  Response to stimulus (GO:0050896) |
| AT1G73080 | Leucine-rich repeat receptor-like protein kinase (PEPR1) | Response to stress (GO:0006950)  Response to stimulus (GO:0050896) |
| AT1G74100 | Sulfotransferase (SOT16) | Response to stress (GO:0006950)  Response to external stimulus (GO:0009605)  Response to wounding (GO:0009611)  Response to endogenous stimulus (GO:0009719)  Response to jasmonic acid (GO:0009753)  Response to stimulus (GO:0050896) |
| AT2G22330 | cytochrome P450, family 79, subfamily B, polypeptide 3 (CYP79B3) | Response to stress (GO:0006950)  Response to external stimulus (GO:0009605)  Response to wounding (GO:0009611)  Response to stimulus (GO:0050896) |
| AT2G28900 | Outer envelope pore protein 16-1, chloroplastic (OEP161) | Response to stress (GO:0006950)  Response to external stimulus (GO:0009605)  Response to wounding (GO:0009611)  Response to endogenous stimulus (GO:0009719)  Response to jasmonic acid (GO:0009753)  Response to stimulus (GO:0050896) |
| AT2G29450 | GSTU5 | Response to stress (GO:0006950) |
|  |  | Response to stimulus (GO:0050896) |
| AT2G29630 | Phosphomethylpyrimidine synthase, chloroplastic (THIC) | Response to external stimulus (GO:0009605)  Response to stimulus (GO:0050896) |
| AT2G32230 | Proteinaceous RNase P 1, chloroplastic/mitochondrial PRORP1) | Response to stimulus (GO:0050896) |
| AT2G34600 | Protein TIFY 5B | Response to endogenous stimulus (GO:0009719)  Response to jasmonic acid (GO:0009753)  Response to stimulus (GO:0050896) |
| AT2G39920 | Uncharacterized protein At2g39920 | Response to stimulus (GO:0050896) |
| AT2G40080 | Protein EARLY FLOWERING 4 (ELF4) | Response to stimulus (GO:0050896) |
| AT2G42530 | Protein COLD-REGULATED 15B, chloroplastic (COR15B) | Response to stress (GO:0006950)  Response to stimulus (GO:0050896) |
| AT2G43550 | Defensin-like protein 197 (ATT1I6) | Response to stress (GO:0006950)  Response to stimulus (GO:0050896) |
| AT4G11360 | E3 ubiquitin-protein ligase (RHA1B) | Response to stimulus (GO:0050896) |
| AT4G16740 | Tricyclene synthase, chloroplastic (TPS03) | Response to stress (GO:0006950)  Response to external stimulus (GO:0009605)  Response to wounding (GO:0009611)  Response to stimulus (GO:0050896) |
| AT4G30650 | UPF0057 membrane protein At4g30650 | Response to stress (GO:0006950)  Response to stimulus (GO:0050896) |
| AT4G30660 | UPF0057 membrane protein At4g30660 | Response to stress (GO:0006950)  Response to stimulus (GO:0050896) |
| AT4G31500 | Cytochrome P450 83B1 (CYP83B1) | Response to stress (GO:0006950)  Response to stimulus (GO:0050896) |
| AT4G31800 | WRKY like transcription factor (WRKY18) | Response to stress (GO:0006950)  Response to stimulus (GO:0050896) |
| AT4G39260 | Glycine-rich RNA-binding protein 8 (RBG8) | Response to stress (GO:0006950)  Response to stimulus (GO:0050896) |
| AT4G39960 | AT4g39960/T5J17_130 | Response to stress (GO:0006950)  Response to stimulus (GO:0050896) |
| AT4G39980 | Phospho-2-dehydro-3-deoxyheptonate aldolase 1, chloroplastic (DHS1) | Response to stress (GO:0006950)  Response to external stimulus (GO:0009605)  Response to wounding (GO:0009611)  Response to stimulus (GO:0050896) |
| AT5G20630 | Germin-like protein subfamily 3 member 3 (GER3) | Response to stress (GO:0006950)  Response to stimulus (GO:0050896) |
| AT5G47240 | nudix hydrolase homolog 8 (atnudt8) | Response to stress (GO:0006950)  Response to external stimulus (GO:0009605)  Response to wounding (GO:0009611)  Response to stimulus (GO:0050896) |
| AT5G54960 | Pyruvate decarboxylase 2 (PDC2) | Response to stress (GO:0006950)  Response to stimulus (GO:0050896) |
| AT5G57110 | Calcium-transporting ATPase (ACA8) | Response to stimulus (GO:0050896) |
| AT5G59080 | Emb | Response to stress (GO:0006950)  Response to stimulus (GO:0050896) |
| AT5G63980 | SAL1 phosphatase (SAL1) | Response to stress (GO:0006950)  Response to endogenous stimulus (GO:0009719)  Response to stimulus (GO:0050896) |
