# Supplementary material for: High-resolution magic angle spinning NMR studies for metabolic characterization of Arabidopsis thaliana mutants with enhanced growth characteristics
Source: PLoS One. 2018 Dec 31;13(12):e0209695. doi: 10.1371/journal.pone.0209695 (PMC6312362; doi:10.1371/journal.pone.0209695)
Supplement: S2 Table — (DOCX) [file pone.0209695.s004.docx]

**S2 Table: Measured metabolites in leaves of Col-0, VP16-02-003 and VP16-05-014 plants and their chemical shift assignment in ^1^H HR-MAS NMR spectrum.**

| **Metabolites** | **Assignment^*^** | **Chemical shift (ppm)** | **Concentrations (% average Col-0)** | |
| --- | --- | --- | --- | --- |
|  |  |  | **VP16-02-003** | **VP16-05-014** |
| L-Alanine (Ala) | ^2^CH | 3.76 | 125.3 ± 5.4 | 104.1 ± 5.9 |
|  | ^3^CH_3_ | 1.46 |  |  |
| β-alanine | ^2^CH_2_ | 2.49 | 179.3 ± 17.2 | 64.0 ± 9.2 |
| L-Asparagine (Asn) | ^2^CH | 3.90 | 64.9 ± 11.0 | 135.1 ± 4.7 |
|  | ^3^CH_2_ | 2.80 |  |  |
| L-Aspartic acid (Asp) | ^2^CH | 4.00 | 75.7 ± 2.9 | 136.1 ± 8.1 |
|  | ^3^CH_2_ | 2.80 |  |  |
| Betaine | ^2^CH_2_ | 3.89 | 75.9 ± 7.0 | 77.1 ± 8.3 |
| Choline | ^1^CH_2_ | 4.05 | 75.2 ± 5.2 | 83.6 ± 6.0 |
|  | ^2^CH_2_ | 3.50 |  |  |
|  | N(CH_3_)_3_ | 3.22 |  |  |
| Fructose | ^3^CH | 4.03 | 51.0 ± 11.4 | 67.8 ± 5.3 |
|  | ^4^CH | 3.89 |  |  |
| Fumaric acid | CH = CH | 6.60 | 119.5 ± 6.3 | 395.8 ± 12.5 |
| Glucose (Glc) | ^2^CH | 3.27 | 51.8 ± 13.6 | 65.0 ± 9.4 |
| L-Glutamic acid (Glu) | ^2^CH | 3.74 | 79.0 ± 5.6 | 72.0 ± 15.0 |
|  | ^3^CH_2_ | 2.12 |  |  |
|  | ^4^CH_2_ | 2.35 |  |  |
| L-Glutamine (Gln) | ^3^CH_2_ | 2.11 | 73.3 ± 7.8 | 99.1 ± 10.8 |
|  | ^4^CH_2_ | 2.43 |  |  |
| L-Glycine (Gly) | ^2^CH_2_ | 3.54 | 76.8 ± 5.4 | 73.3 ± 7.5 |
| Lactic acid | ^2^CH | 4.10 | 149.1 ± 13.1 | 66.8 ± 7.3 |
|  | ^3^CH_3_ | 1.32 |  |  |
| L-Lysine (Lys) | ^6^CH_2_ | 2.96 | 78.4 ± 6.4 | 98.7 ± 11.8 |
| Malic acid | ^2^CH_2_ | 4.29 | 51.8 ± 13.0 | 62.4 ± 8.5 |
|  | ^5^CH_2_ | 2.64 |  |  |
|  | ^5`^CH_2_ | 2.50 |  |  |
| Myo-inositol | ^1^CH | 3.52 | 71.7 ± 9.7 | 48.3 ± 15.6 |
|  | ^2^CH | 4.05 |  |  |
| Phenylalanine (Phe) | ^4^CH | 7.36 | 65.2 ± 10.6 | 144.8 ± 8.0 |
|  | ^6^CH | 7.32 |  |  |
| L-Tyrosine (Tyr) | ^2^CH | 7.19 | 62.1 ± 20.6 | 48.6 ± 33.2 |
|  | ^3^CH | 6.89 |  |  |

*Assignment according to ref [15]
